# Cross-calibration of the Siemens mMR: easily acquired accurate PET phantom measurements, long-term stability and reproducibility

**DOI:** 10.1186/s40658-016-0146-3

**Published:** 2016-07-07

**Authors:** Sune H. Keller, Björn Jakoby, Susanne Svalling, Andreas Kjaer, Liselotte Højgaard, Thomas L. Klausen

**Affiliations:** 3982 Department of Clinical Physiology, Nuclear Medicine and PET, Rigshospitalet (University of Copenhagen), Blegdamsvej 9, DK-2100 Copenhagen, Denmark; Diagnostic Imaging, Magnetic Resonance, Siemens Healthcare GmbH, Allee am Roethelheimpark 2, 91052 Erlangen, Germany; University of Surrey, Guildford, UK

**Keywords:** PET/MR, Quality control, Cross-calibration, Calibration, PET phantom

## Abstract

**Background:**

We present a quick and easy method to perform quantitatively accurate PET scans of typical water-filled PET plastic shell phantoms on the Siemens Biograph mMR PET/MR system.

We perform regular cross-calibrations (Xcal) of our PET systems, including the PET/MR, using a Siemens mCT water phantom.

**Long-term stability:**

The mMR calibration stability was evaluated over a 3-year period where 54 cross-calibrations were acquired, showing that the mMR on average underestimated the concentration by 16 %, consistently due to the use of MR-based μ-maps.

The mMR produced the narrowest calibration ratio range with the lowest standard deviation, implying it is the most stable of the six systems in the study over a 3-year period.

**mMR accuracy with predefined μ-maps:**

With the latest mMR software version, VB20P, it is possible to utilize predefined phantom μ-maps. We evaluated both the system-integrated, predefined μ-map of the long mMR water phantom and our own user-defined CT-based μ-map of the mCT water phantom, which is used for cross-calibration.

For seven scans, which were reconstructed with correctly segmented μ-maps, the mMR produced cross-calibration ratios of 1.00–1.02, well within the acceptance range [0.95–1.05], showing high accuracy.

**Conclusions:**

The mMR is the most stable PET system in this study, and the mean underestimation is no longer an issue with the easily accessible μ-map, which resulted in correct cross-calibration ratios in all seven tests. We will share the user-defined μ-map of the mCT phantom and the protocol with interested mMR users.

## Findings

### Introduction

Accurate PET measurements on PET/MR systems are problematic with MR-based attenuation correction (MRAC) [9, 11, 12]. For phantoms, the plastic materials in MR-based μ-maps are typically segmented as air and are assigned with a linear attenuation coefficient (LAC) of 0 cm^−1^ when they ought to have LACs close to that of water [2, 11, 12].

For single phantom scans, one can utilize externally acquired, co-registered, and converted (Hounsfield units (HU) to 511 keV LACs) CT-based μ-maps [8] or calculated μ-maps [4]. This approach is time-consuming and error-prone, and it requires either the use of external μ-maps or access to an external reconstruction setup. Thus, it is not a suitable approach for regularly repeated phantom scans, e.g., for routine quality control (QC) such as our regularly performed cross-calibrations (Xcal) [1, 6].

**Fig. 1:**
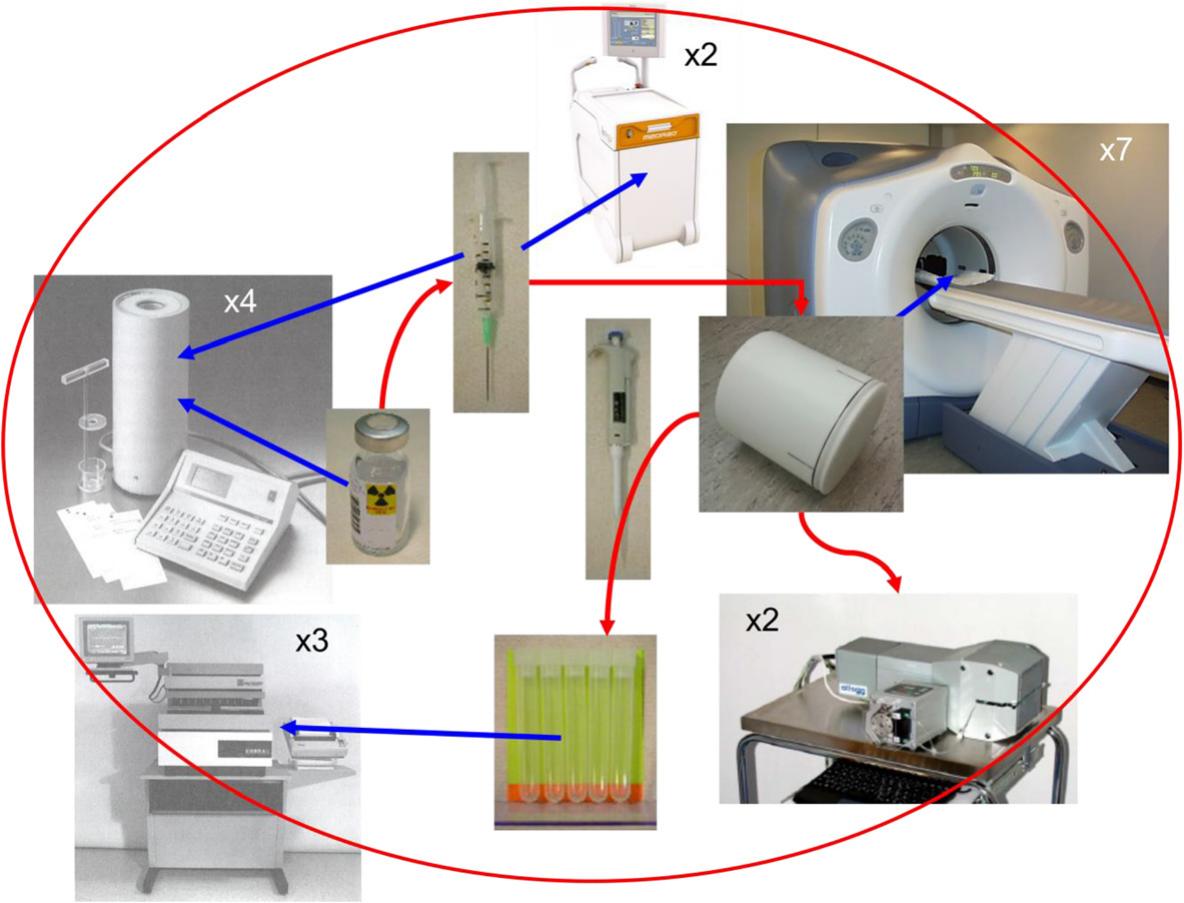
Cross-calibration procedure. To ensure quantitatively accurate measurements of activities at our PET center, we perform a quality control of our devices every 2–3 weeks, testing they are calibrated to measure within our acceptance range. Since this quality control (QC) procedure cross-checks the calibration of all devices compared to a reference device, a Veenstra VDC-404 dose calibrator, it is denoted a cross-calibration [1, 6]. We include all devices in use, which as of 2015 are as follows: one PET/MR, five PET/CT systems (one is exclude in our 3-year comparison as it has only been installed for 2 of the 3 years), one HRRT PET system, three well counters, two blood samplers, two automatic injectors, and four dose calibrators (incl. the reference). Each device measures the original dose, the filled phantom, or samples drawn from the phantom as illustrated above

Cross-calibration is a quality control procedure to check the quantitative accuracy of our systems (PET, PET/CT, and PET/MR) and other equipment (Fig. 1).

In the current work, we firstly present a quick and easy method to perform quantitatively accurate PET scans of a typical water-filled plastic shell cylinder phantom on the Siemens mMR PET/MR system. We describe how to integrate an external, CT-based μ-map into the system software as a user-defined μ-map for routine use.

As a second part of the assessment of the mMR phantom scan performance, we evaluate the results of executing cross-calibrations every 2–3 weeks over a 3-year period, comparing the mMR to five other PET systems to assess the long-term stability and reproducibility.

### Material and methods

**Fig. 2:**
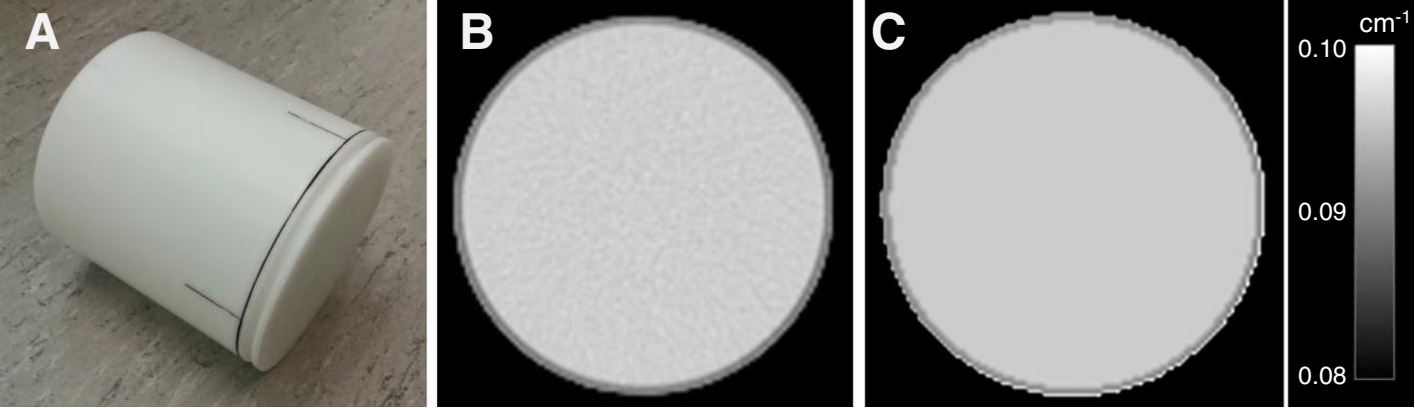
The phantom used for cross-calibration scans. **a** The Siemens mCT water phantom of 20 cm inner diameter and axial length, 21 cm outer diameter, and 6283 ml volume. **b** CT scan of the phantom after conversion to linear attenuation coefficient map (μ-map). **c** The calculated μ-map of the water phantom supplied with the mMR. We can use the μ-map in **c** directly as we scan the mCT water phantom in the default position of the mMR water phantom, which is made of the same material and has the same inner and outer diameters as the mCT phantom in **a** but is 10 cm longer to cover the longer PET FOV of the mMR. The μ-map in **c** is given as a predefined μ-map in the mMR system reconstruction software (version VB20P)

The PET/MR system under evaluation is the Siemens Biograph mMR [2, 4] at Rigshospitalet, Copenhagen. The cross-calibration phantom we used in this study was a Siemens mCT water phantom (Fig. 2a), for which we had acquired a CT scan on a Siemens Biograph mCT PET/CT system (Fig. 2b) [7].

All PET scans on the mMR were acquired for 5 min with the phantom placed in the lowest vertical position on the mMR phantom holder and positioned axially using the positioning laser and scanning with a fixed protocol to ensure we always scanned in the same position. The mCT phantom was scanned with varying concentrations of [^18^F]-FDG in water (see Table 1).

**Table 1:** Test of μ-maps for mMR cross-calibration: concentrations (kBq/ml) and ratios

μ-map	Reference concentration	mMR concentration	mMR Xcal concentration ratio
CT-based (+8 mm in *x*)	10.77	10.79	1.00
CT-based (−10 mm in *x*)	10.77	10.79	1.00
CT-based (−2 mm in *x*)	18.78	19.04	1.01
CT-based (no shift)	11.29	11.57	1.02
mMR water (original)	11.86	11.96	1.01
mMR water (original)	10.77	10.91	1.01
mMR water (original)	11.29	11.55	1.02

The mMR PET images were reconstructed on the mMR using OP-OSEM with 4 iterations, 21 subsets, and a 3-mm FWHM Gaussian post-reconstruction filter into 344 × 344 × 127 matrices of 0.83 × 0.83 × 2.03 mm^3^ voxels.

We used a Veenstra VDC 404 dose calibrator as reference, and all devices in the cross-calibration should measure the same concentration within ±5 % of the reference concentration, i.e., have Xcal concentration ratios in the range [0.95–1.05].

#### Predefined μ-map options as alternatives to the standard Dixon MR-based μ-maps

The mMR software (version VB20P, available since Q4 2013) offers the choice of four easily accessible predefined μ-maps for phantom reconstructions as alternative to the standard Dixon MR-based μ-maps. Two of the predefined choices are user-defined options, which we use to test our external CT-based μ-map. As an alternative, we also tested the μ-map of the mMR water phantom, which is given as one of the two predefined and build-in μ-maps (Fig. 2c): The mCT and mMR water phantoms are made of the same material and have the same diameter and wall thickness (see Fig. 2). We acquired and reconstructed seven PET scans on the mMR of the mCT phantom using five different predefined μ-maps. In three cases, the μ-maps were shifted up to 10 mm in the *x*-*y* plane testing robustness against misregistrations.

#### Creation of a user-defined CT-based μ-map

After a 120-kVp CT scan of the mCT phantom, the CT image was automatically registered to an mMR PET scan of the mCT phantom using Vinci 2.55 [10]. HU were converted to linear attenuation coefficients at 511 keV in Matlab following the method of Carney et al. [3], where LAC = 9.6× 10^5^ × (HU + 1000). A 3-mm filter was applied in Vinci, and images were saved in interfile format. Header text files were then generated according to the specifications in [5]. The registration procedure above served mostly to correct any rotational differences because the translational positioning of the μ-map had to be set in the header, specifying a μ-map origin in pixels and an origin offset in millimeters relative to the system patient table origin. This requires fixed phantom positioning for all PET scans performed using this μ-map. Finally, the image and the corresponding header were named User_Defined_n.v(.hdr) (*n* = 1, 2) and saved in a dedicated folder for predefined μ-maps.

#### Routine cross-calibration

The mMR has been included in our routine cross-calibration procedure since its installation but has not yielded concentration ratios in the ±5 % acceptance range while using MR-based μ-maps, which segments plastic as air. Even though the mMR PET images using MR-based μ-maps are incorrectly quantified, they are consistently (and reproducibly) erroneous, meaning we can still use the cross-calibration results to evaluate the stability of the system in comparison with our five other PET systems (details in Table 2) included in the cross-calibration during the same 3-year period (02/2012–01/2015). Since the mMR has been calibrated three times using a correct μ-map (and a complex procedure) in the 3-year period, we are certain that it measures accurately and the consistent bias is purely due to the incorrect MR-based μ-maps.

**Table 2:** Statistics of the Xcal ratios for six systems over 3 years

System	Mean	SD	Range [min–max]
PET1: Siemens HRRT	0.99	0.0269	[0.94–1.05]
PET3: Siemens Biograph mCT	1.00	0.0266	[0.95–1.04]
PET4: Siemens Biograph mCT	1.01	0.0265	[0.95–1.06]
PET5: Siemens Biograph True-Point TrueV	1.01	0.0238	[0.95–1.06]
PET6: Siemens Biograph True-Point TrueV	1.00	0.0209	[0.95–1.04]
PET7: Siemens Biograph mMR	0.84	0.0143	[0.81–0.88]

All measurements of the concentrations in the images were performed in Siemens TrueD using a cylindrical volume of interest (VOI) placed centrally in the phantoms, 12–15 cm in diameter and 500–950 cm^3^ in volume.

### Results

#### Test of μ-maps

**Fig. 3:**
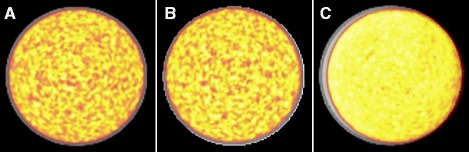
Fusion of μ-maps and mMR cross-calibration PET scans of the mCT phantom. **a** User-defined CT-based (no shift) μ-map. **b** Predefined original mMR water phantom μ-map. **c** CT-based μ-map with an 8-mm shift (misregistration)

We acquired and reconstructed seven PET scans on the mMR of the mCT phantom using five different μ-maps (Table 1). All images resulted in Xcal ratios well within the acceptance range [0.95–1.05] as reported in Table 1. Examples of fused images are shown in Fig. 3.

**Fig. 4:**
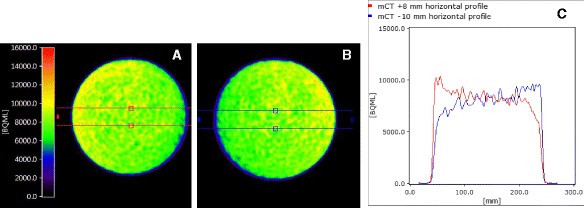
Gradient effect in PET quantification caused by μ-map shifts (misregistrations). PET images of the mCT water phantom with an 8-mm horizontal shift of the μ-map (**a**) and a 10-mm opposite shift (**b**). **c** The horizontal profile plots (the *red* and *blue overlays* in **a** and **b**) through the PET images

As it took a few cycles to get the translational position of the user-defined μ-map set correctly in the image header, we found that obtaining the Xcal ratios in the center of the phantom is robust against misregistrations of up to 10 mm on the *x*-axis (results in Table 1). Plotting horizontal profiles through the image slices showed a clear gradient across the mCT phantom in the *x*-direction when using the μ-maps shifted by 8 and 10 mm as seen in Fig. 4. Therefore, only cylindrical VOIs placed balanced around the center guarantees valid measurements with these shift magnitudes. When using the 2-mm shifted μ-map or the original mMR water phantom μ-map with a 1–2-mm shift in the *x*-*y* plane (Fig. 3b), a very weak, negligible gradient is observed. Although, the predefined μ-map of the mMR water phantom is longer and thus matches a *z*-position, which is different than that of the shorter mCT phantom, it can still be used as a μ-map for the mCT phantom; VOIs in the central parts of the reconstructed PET images are not affected.

#### Long-term mMR calibration stability

**Fig. 5:**
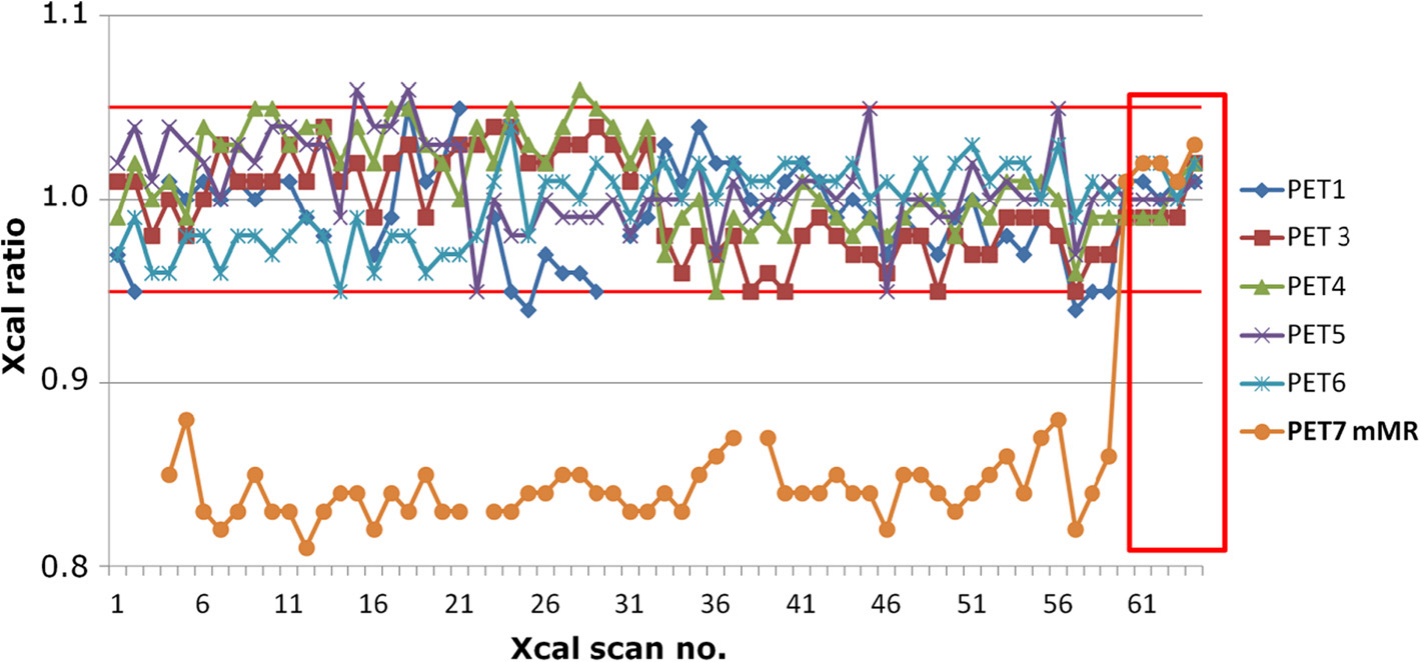
Cross-calibration ratios for six PET systems. The 59 cross-calibrations included in this study are plotted outside the *red box* and show high long-term stability and reproducibility of all scanners: Only five Xcal ratios were outside the ±5 % acceptance range indicated by the *red horizontal lines* and only by 1 % each of the five times (and never twice in a row). In the *red box*, five cross-calibrations using the user-defined μ-map for the mMR PET/MR system are plotted, showing the accuracy of the mMR in the period February–May 2015 (following directly after our 3-year study and initial testing of the predefined μ-maps). PET1 (HRRT) and the mMR were each left out 5 of the 59 times (details for the mMR given in the text, the HRRT because of 1–2-week setups were ongoing or other unavailability)

We performed 59 cross-calibrations over a 3-year period, with the mMR left out five times: once, the system was unavailable, and at four occasions, the MR-based μ-map was accidentally reconstructed (irreversibly) without the two-compartment phantom segmentation option selected, yielding a μ-map with the water segmented as fat (LAC = 0.085 cm^−1^ instead of 0.096 cm^−1^) causing Xcal ratios at around 0.70. Figure 5 shows all included measurements on the six systems over the 3-year period.

Table 2 shows that the mMR measures activity concentrations are 16 % too low on average due to the use of MR-based μ-maps with the plastic body of the phantom segmented as air. All other systems measure very close to 1.00 on average. We only had ratios out of the acceptance range in 5 out of 290 scans (PET1: 2× 0.94, PET4: 1× 1.06, and PET5: 2× 1.06) and newer twice in a row (see Fig. 5).

### Discussion

The results in Table 2 show that the mMR has a smaller standard deviation (SD) and narrower range of cross-calibration ratios than the other systems, which implies high reproducibility. Scaling the 54 mMR Xcal ratios reconstructed with the MR-based μ-map to a mean of 1.00 by dividing each by the actual mean (0.84) changes the range to [0.97–1.05], which is still narrower than for any other systems in this study, and the SD changes to 0.0170 (still the lowest). Thus, the mMR is the most stable of the systems over a 3-year period, which could be caused by its use of avalanche photodiode (APD) PET detectors instead of conventional photomultiplier tubes (PMTs).

The long-term stability and accuracy of all six PET systems in this study were high with only 5/290 measures out of range (off by 1 % each time and newer off twice in a row) warranting no further actions to ensure the systems measure accurately. The correction would be to redetermine the ECAT calibration factors (ECFs) normally only adjusted when a new [^68^Ge]-phantom for daily QC, normalization, and setup is put in use (at 1.5-year intervals).

This phantom study is limited to cross-calibrations using [^18^F]-FDG. But the μ-maps used are tracer-independent, and similar μ-maps could be generated for other phantoms scanned on a regular basis in a fixed position.

### Conclusion

Over a 3-year period and 54 cross-calibrations, the mMR showed to be the most stable of the six PET systems evaluated in this study. The Xcal ratios were persistently off by a factor of 16 % due to the use of MR-based μ-maps, a factor that we can now easily eliminate by using correct μ-maps.

We have successfully demonstrated a procedure to perform accurate cross-calibration of the mMR PET/MR system. Both a new CT-based user-defined μ-map of the mCT water phantom and a predefined μ-map of the mMR water phantom resulted in accurate cross-calibration ratios. The μ-maps are available as an easily accessible drop-down option in the system’s user interface.

We will share our user-defined μ-map of the mCT phantom and the protocol with interested mMR users, who wish to employ our method. Following our work, one can also generate user-defined μ-maps for other frequently used phantoms. If compliant with local procedures, the mMR water phantom can also be used across systems for cross-calibrations.
